# Suicidal ideation among recently arrived refugees in Germany

**DOI:** 10.1186/s12888-022-03844-z

**Published:** 2022-03-15

**Authors:** Yuriy Nesterko, Elisa Haase, Antje Schönfelder, Heide Glaesmer

**Affiliations:** Department of Medical Psychology and Medical Sociology, University of Leipzig, Philipp-Rosenthal-Str. 55, 04103 Leipzig, Germany

**Keywords:** Suicidal ideation, Refugees, Traumatic experiences, Mental health

## Abstract

**Background:**

Refugees are considered a high-risk population for developing mental health disorders. Yet little research has been conducted on suicidal ideation among refugees resettled in Western high-income countries. In the present hstudy, suicidal ideation and its association with different socio-demographic, flight-related, and mental health-related factors were analyzed in recently arrived refugees in Germany.

**Methods:**

The study was conducted in a reception facility for asylum-seekers in Leipzig, where 564 newly arrived adult residents participated. The questionnaire included socio-demographic and flight-related questions as well as standardized instruments for assessing suicidal ideation (item 9 from PHQ-9), a variety of traumatic experiences (LEC-5), posttraumatic stress disorder (PCL-5), depression (PHQ-8), and somatic symptoms (SSS-8). Multiple logistic regression models were run to predict suicidal ideation in relation to different socio-demographic, flight, and mental health-related factors.

**Results:**

In total, 171 (30.3%) participants who had just or very recently arrived in Germany reported having experienced suicidal ideation within the two weeks prior to being assessed. Those who reported suicidal ideation also reported higher prevalence of somatic symptoms, posttraumatic stress disorder, depression, and experiences of sexual violence, as well as worse self-rated mental and physical health. In addition, there were significant independent associations between suicidal ideation and (1) younger age, (2) longer flight duration, (3) experiences of sexual violence, (4) symptoms of posttraumatic stress disorder, and (5) symptoms of depression.

**Conclusions:**

The results emphasize the association between suicidal ideation and different clinically relevant mental health symptoms among newly arrived refugees in Germany. Special attention should not only be given to refugees suffering from symptoms of poor mental health, but also to those of younger age as well as refugees who have experienced sexual violence, as they might be affected by suicidal ideation whether or not they suffer from other mental health problems.

## Background

The number of people who have been forced to leave their homes has been growing dramatically for years. In the last ten years in particular, the increase has been enormous – from 2010 to 2020, the number of forcibly displaced people worldwide doubled from around 41 million to more than 82 million, as has been reported annually by the United Nations High Commissioner for Refugees. In 2020, the vast majority, 48 million people, were internally displaced, followed by 26.4 million refugees, 4.1 million asylum-seekers, and 3.9 million Venezuelans displaced abroad [1]. Many of those millions, and especially those who are refugees, have experienced a wide spectrum of adverse and/or stressful events before, during, and/or after fleeing and are consequently considered a high-risk population for developing mental disorders [2, 3]. Indeed, many refugees regularly face stressful and traumatizing experiences such as exposure to armed conflict, genocide, imprisonment, torture, sexual violence, life-threatening journeys, separations, lack of food, water or shelter, witnessing death, detention, ethnic and/or racial discrimination, as well as restricted access to health care, education and/or work, many of which are considered human rights violations [4, 5]. In light of such experiences, most refugees are dealing with various severe losses (family members, friends, social status, language, etc.) and grief processes that directly or indirectly threaten their mental health, regardless of whether they are presently safe or not [6]. Available evidence on mental health in refugees generally concentrates on (1) prevalence rates of different mental disorders, (2) risk factors for developing mental disorders with respect to the process of fleeing, and (3) development and/or evaluation of treatment programs for those in need, with the greatest attention and effort invested so far in research on posttraumatic stress disorder (PTSD) and depression among different groups of refugees [2].

By contrast, there is little evidence on suicidal ideation and behavior among this population, although, in general, the link between suicidality, trauma exposure, and mental health problems is well-known [6, 7]. In the present study, we focus on suicidal ideation and its association with different socio-demographic, flight related, and mental health-related factors among recently arrived refugees in Germany.

The global lifetime prevalence of suicidal ideation is 9.2% across 17 countries in Africa, Asia, the Pacific, North America, Europe, and the Middle East, revealing a range of 3.1–12.0% in developing countries and 3.0–15.9% in developed countries [8]. General risk factors for suicidal ideation include: previous suicidal ideation and behavior, feelings of hopelessness, symptoms of depression, anxiety, abusive experiences in childhood [9], somatic pain, [10] and female sex [8, 11]. As mentioned above, there are few studies on suicidality in general and suicidal ideation in particular among refugee populations, and those that do exist demonstrate rather contradictory results. Whereas some recently published studies from Sweden report similar or a lower suicide risk among different groups of refugees compared to Swedish-born majority population [12, 13], others from the Netherlands [14, 15] and the UK [16] show higher rates of suicidal behavior among refugees compared to natives. In a study on suicidal ideation (during the month previous to assessment) among refugees in Nigeria, 27.3% of the participants reported suicidal ideation, revealing a significantly higher prevalence rate compared to host country residents (17.3%) [17]. Two studies conducted in European initial reception facilities for asylum-seekers reported prevalence rates for suicidal ideation during the preceding two weeks of 33.9% (*n* = 510) among refugees from Afghanistan and Syria [18] hosted in Sweden, and 5.6% (*n* = 209) among a heterogenous group of refugees living in Germany [19]. More generally, evidence on suicidal ideation in cross-national comparison indicates a link between suicidal ideation and exposure to specific traumatic events, [20, 21] the strongest associations being with sexual and/or interpersonal violence regardless of the presence of PTSD. For example, in a study by Ferrada-Noli et al. [22], a relationship between suicidal ideation and experiences of torture were found in a heterogenous sample of refugees, and there are several studies showing a link between suicidal ideation and experiences of sexual violence in female [23, 24] and male [25] refugees. Furthermore, in a study on war-related traumatic experiences and suicidal ideation and behavior among 854 refugees from former Yugoslavia living in Germany, Italy, and the UK, the relationships between suicidal ideation and behavior and war-related traumatic experiences such as imprisonment were significant, even after controlling for common mental disorders (PTSD, major depression, psychotic disorders and substance use disorders) several years after a person’s exposure to a traumatic experience [26]. To sum up, the limited evidence currently available suggests that suicidal ideation among refugees is a common phenomenon that is associated with flight and/or war-related traumatic experiences, irrespective of the presence of mental disorders [21, 25, 26].

As there is only little research on suicidal ideation among refugees currently living in Germany, the host country with the highest number of refugees in Europe [1], the aim of the present study was to analyze the prevalence of suicidal ideation and its associations with different (1) socio-demographic characteristics, (2) flight and war-related traumatic experiences (e.g., combat exposure, captivity), (3) experiences of sexual violence, and (4) different health outcomes (e.g., symptoms of depression, symptoms of PTSD, somatic symptoms) in refugees of different origins upon their arrival in Germany, based on survey data collected in an epidemiological study.

## Methods

### Data collection and study sample

The original study conducted to assess the prevalence of common mental health disorders in newly arrived refugees with an epidemiological approach was run between May 2017 and June 2018 at an initial reception facility operated by the Federal State of Saxony for asylum-seekers in Leipzig, Germany [3; 5]. The study’s target population consisted of adult individuals (≥18 years) who were currently residing at the facility during the survey period. Usually, refugees arriving in the facility apply for asylum within the first two weeks. Thus, all participants in the present study did officially became asylum-seekers, but, depending on the exact date of their participation, some of them did not yet have this status when they were assessed. Thus, we use the term ‘refugees’ to describe all of the participants.

Based on the facility’s registration data of all newly arrived residents, potential study participants were approached by members of the project staff in their accommodation unit, informed about the study objectives as well as the data protection policy, and, in the event that they were willing to participate, introduced to the survey procedure. Between May 1st and May 15th of 2017, the participants were asked to fill out a paper version of the questionnaire (pilot study; *N* = 67); after May 17th of 2017, the participants filled out a tablet-based questionnaire in their respective native language [3; 5]. After receiving information sheets and providing informed consent, the participants responded to the questionnaire on their own (time needed approximately: 45 min). Project staff was available to answer questions when necessary. The assessments took place three times a week, on Mondays, Wednesdays, and Thursdays between 10 a.m. and 1 p.m. Data were electronically transferred and administered consecutively to the ongoing data collection platform using LimeSurvey Offline-App for android systems. Data control and consistency checks were carried out at monthly intervals and a simple plausibility check was carried out immediately after the entry of a maximum of 30 data sets. Data were stored in anonymous form on a computer at the University of Leipzig network in accordance with the data protection guidelines [3; 5].

A total of 1316 adult individuals were newly accommodated at the primary reception facility during the survey period, 569 of whom took part in the study (response rate 43.2%). Of these, 67 individuals completed the paper version of the questionnaire and 502 responded via tablet. Generally, about 60% (*n* = 297) were assessed during the first seven days after the arrival, another 19.9% (*n* = 100) during the second week (between 8 and 14 days) after the arrival, 10% (*n* = 50) during the period of 15–28 days after the arrival, and finally 10.9% (*n* = 55) > 28 days after the arrival [3; 5]. Data on all non-participants’ age, sex, and country of origin were recorded to identify possible selection bias. Detailed information on age, sex, and country of origin of non-participants as well as calculated non-response weights were published previously [3]. For the present analyses, we used data from 564 newly arrived residents with valid information about suicidal ideation.

The study was approved by the Ethics Committee of the Medical Faculty of the University of Leipzig (446/16-ek). All study procedures were conducted in accordance with the Helsinki Declaration and its later amendments or comparable ethical standards. Written informed consent was granted by all study participants.

### Assessments

The questionnaire used in the study included (1) socio-demographic and flight-related questions, (2) standardized instruments for assessing PTSD, depression, and somatic symptoms, as well as (3) questions assessing self-rated mental and physical health. The German version of the questionnaire was translated and back-translated into 10 different languages (Albanian, Arabic, English, Farsi, French, Kurdish, Russian, Spanish, Turkish, and Urdu) by a professional translation agency specialized in medical translations [3; 5]. The languages included were chosen based on the proportion of refugees who had arrived in Germany during the year preceding the survey. The Tigrinya version of the questionnaire was translated and back-translated by the same agency based on the English version of the questionnaire. All back-translations were reviewed by the first and last author and, when necessary, returned to the agency for final modifications [3; 5].

### Sociodemographic and flight-related characteristics

Participants were asked to provide information about their age, sex, country of origin, marital status, number of children, level of education, accompaniment during their flight, as well as current information about family and friends left behind [3].

### Suicidal ideation and depression

Item 9 of the Patient Health Questionnaire (PHQ-9) depression module (“Thoughts that you would be better off dead, or of hurting yourself in some way”) [27] was used to assess suicidal ideation. The PHQ-9 contains nine items rated on a scale of 0 (‘not at all’) to 3 (‘nearly every day’) which reflects the frequency with which participants have experienced the symptom in question within the previous 2 weeks. In the present study, those who indicated 0 (‘not at all’) while responding to item 9 were classified as participants without suicidal ideation, all other participants indicating 1 (‘several days’) to 3 (‘nearly every day’) were classified as participants with suicidal ideation. The other 8 Items were used to assess depression. As suggested by Kroenke et al. [28], participants with PHQ-8 score of > 9 were classified as participants exceeding the threshold for a positive diagnosis of depression. Cronbach’s α of PHQ-9 in the present study was α = .84 (.70 to .89 for the different language versions).

### Sexual violence

Experiences of sexual violence (lifetime) were assessed with two items from the DSM-5 Life Events Checklist (LEC-5) [29] “Sexual assault (rape, attempted rape, being forced to perform any type of sexual act by force or threat of force) “ and/or “Other unwanted sexual experiences” with the response options “happened to me” and/or “witnessed it.”

### Combat or exposure to a war-zone

Traumatic experiences related to combat or exposure to a war-zone in the military or as a civilian (lifetime) were assessed using the DSM-5 Life Events Checklist (LEC-5) [29] Item 10 “Combat or exposure to a war-zone (in the military or as civilian)” with the response option “happened to me.”

### Captivity

Experiences of captivity (lifetime) were assessed using the DSM-5 Life Events Checklist (LEC-5) [29] Item 11 “Captivity (for example, being kidnapped, abducted, held hostage, prisoner of war)” with the response option “happened to me.”

### Post-traumatic stress disorder

PTSD was assessed with the PCL-5 (PTSD-Checklist), a 20-item self-report instrument, which assesses symptoms of PTSD as defined by the DSM-5 [30]. The 20 items of the PCL-5 reflect the frequency with which respondents have experienced the item in question rated on a 5-point Likert-scale ranging from ‘not at all’ to ‘extremely.’ A total score (0–80) can be obtained by summing up the scores of each of the 20 items. A score at or above the cut-off score of 33 indicates the presence of PTSD in the respondent. Cronbach’s’ α in the present study was α = .95 (.93 to .97. for the different language versions) [3; 5].

### Somatic symptoms

Somatic symptoms were assessed with the Somatic Symptom Scale-8 (SSS-8) [31]. The SSS-8 is a shortened version of the PHQ-15 questionnaire developed for DSM-5 field trials. Each item can be rated on a 5-point Likert-Scale from ‘not at all’ to ‘very much’ referring to the previous 7 days. The total scores therefore range from 0 to 32, and are subdivided into five categories of severity: ‘none to minimal’ (0–3), ‘low’ (4–7), ‘medium’ (8–11), ‘high’ (12–15), and ‘very high’ (16–32) somatic symptom burden. A cut-off score of > 11 was used for the present study. The internal consistency was α = .84 (.77 to .93 for the different language versions) [3; 5].

### Self-rated mental and physical health status 

Participants were asked to rate their current mental and physical health status on a visual analog scale ranging from 0 to 100, with higher scores indicating better health status [3].

### Statistical analyses

Statistical analyses were performed using the IBM SPSS statistical package, version 27.0 for Windows. Frequencies, means, and standard deviations were used to characterize the study sample. The symptom burden levels of the mental disorders investigated were calculated according to the cut-off scores of each questionnaire. To compare potential differences between the subgroups *t*-tests and *χ*^2^-tests were calculated. Logistic regression model was run to look for possible associations of suicidal ideation with different socio-demographic, flight-related, and mental health outcomes.

## Results

**Fig. 1:**
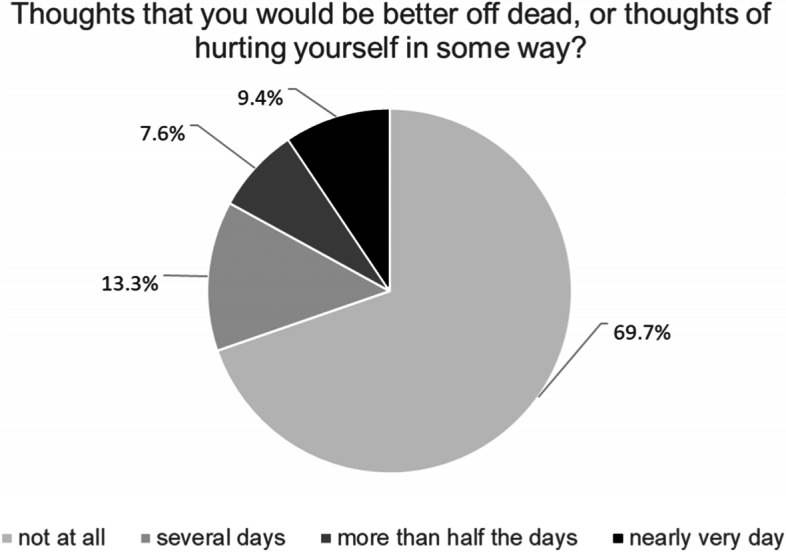
Suicidal ideation among newly arrived refugees in Germany within the last two week

In total, 171 (30.3%) participants reported having experienced suicidal ideation in the preceding two weeks, 75 (43.9%) on several days, 43 (25.1%) on more than half of the days, and 53 (31%) nearly every day (Fig. 1). Participants’ socio-demographic and flight-related characteristics are displayed in total and stratified by suicidal ideation in Table 1.

**Table 1:** Sociodemographic and flight-related characteristics of the study sample

	Participants with suicidal ideation ***N*** = 171 (30.3%)	Participants without suicidal ideation ***N*** = 393 (69.7%)	***χ***^***2***^ ***/ t***	Total ***N*** = 564
**Age**				
M/SD/Range	28.40/8.00/18–61	30.76/9.32/18–70	2.886**	30.04/9.00/18–70
18–29 years 30–39 years 40–49 years > 50 years	116 (67.8%) 40 (23.4%) 11 (6.4%) 4 (2.3%)	209 (53.2%) 121 (30.8%) 40 (10.2%) 23 (5.9%)	11.646**	325 (57.6%) 161 (28.5%) 51 (9.1%) 27 (4.8%)
**Sex**				
female male	53 (31%) 118 (69%)	120 (30.5%) 273 (69.5%)	.012	171 (30.3%) 393 (69.7%)
**Country of origin**				
Cameroon Eritrea Iraq Libya Nigeria Syria Turkey Venezuela other^a^	31 (18.1%) 10 (5.8%) 12 (7%) 11 (6.4%) 17 (9.9%) 8 (4.7%) 10 (5.8%) 12 (7%) 60 (35.1%)	61 (15.5%) 39 (9.9%) 15 (3.8%) 13 (3.3%) 20 (5.1%) 47 (12%) 43 (11%) 73 (18.6%) 82 (20.8%)	83.259***	92 (16.3%) 49 (8.7%) 27 (4.8%) 24 (4.3%) 37 (6.6%) 55 (9.8%) 53 (9.3%) 85 (15.1%) 142 (25.1%)
**University degree**^**1**^				
yes no	69 (40.6%) 101 (59.4%)	207 (52.9%) 184 (47.1%)	7.234**	276 (49.2%) 285 (50.8%)
**Marital status**				
single married divorced widowed	111 (64.9%) 46 (26.9%) 8 (4.7%) 6 (3.5%)	211 (53.7%) 156 (39.7%) 21 (5.3%) 5 (1.3%)	11.233*	322 (57.1%) 202 (35.8%) 29 (5.1%) 11 (2.0%)
**Partnership**				
yes no	50 (29.2%) 121 (70.8%)	161 (41%) 232 (59%)	6.998**	211 (37.4%) 353 (62.6%)
**Parenthood**				
yes no	62 (36.3%) 109 (63.7%)	159 (40.5%) 234 (59.5%)	.882	221 (39.2%) 343 (60.8%)
**Accompaniment during the flight**				
alone strangers friends family members	81 (47.4%) 43 (25.1%) 16 (9.4%) 31 (18.1%)	170 (43.3%) 97 (24.7%) 40 (10.2%) 86 (21.9%)	1.353	251 (44.5%) 140 (24.8%) 56 (9.9%) 117 (20.7%)
**Current information about family left behind**				
yes no	76 (44.4%) 95 (55.6%)	234 (59.5%) 159 (40.5%)	10.972**	310 (55%) 254 (45%)
**Flight duration (in years)**^**2**^				
M/SD/Range	2.53/4.04/0–27	1.65/2.51/0–24	−3.081**	1.91/3.06/0–27

*p < .05; **p < .01; ***p < .001; ^1^N = 561; ^2^N = 540; ^a^ Country of origin other (N for total): Afghanistan (11), Algeria (4), Armenia (3), Belarus (1), Colombia (1) Ethiopia (19), Ghana (3), Georgia (9), Greece (2), India (2), Iran (7), Jordan (2), Kosovo (1), Kuwait (1), Lebanon (7), Liberia (1), Morocco (4), Myanmar (3), Palestine (13), Pakistan (7), Russian Federation (12), Senegal (2), Somalia (7), Sri Lanka (1), Tunisia (7), Ukraine (1), stateless (11)

The mean age of the participants was 30.04 (SD = 9.00) years, indicating significantly younger age in participants with suicidal ideation (*χ*^*2*^(3, 564) = 11.646, *p* < .01; e.g., 67.8% vs. 53.2% for age group 18–29 years). More than two-thirds of the participants were male, with no significant differences between the subsamples with and without suicidal ideation. Most participants originated from Cameroon (*n* = 92, 16.3%), Venezuela (*n* = 85, 15.1%), and Syria (*n* = 55, 9.8%), with significant differences between the subsamples with and without suicidal ideation (*χ*^*2*^(36, 564) = 83.259, *p* < .001; e.g., 4.7% vs. 12% for Syria). All in all, participants from over 30 different countries took part in the survey. The mean flight duration – time period between leaving the home country and arrival in Germany – was 1.91 (SD = 3.06) years in total, with longer duration in participants with suicidal ideation (*t*(538) = − 3.081, *p* < .01; 2.53 vs. 1.65). A bit less than a half of the participants (*n* = 276, 49.2%) reported having a university degree, with significant differences between the subsamples (*χ*^*2*^(1, 561) = 7.234, *p* < .01; e.g., 40.6% of participants with vs. 52.9% of participants without suicidal ideation). A total of 322 (57.1%) participants were single, 202 (35.8%) were married, 29 (5.1%) divorced, and 11 (2%) widowed, also significantly differing between the subgroups (*χ*^*2*^(3, 564) = 11.233, *p* < .05; e.g., 64.9% vs. 53.7% of single participants with vs. without suicidal ideation). In addition, significant differences between the subsamples were found for partnership status (*χ*^*2*^(1, 564) = 6.998, *p* < .01) as well as whether the participants had current information about family members left behind (*χ*^*2*^(1, 564) = 10.972, *p* < .01), whereby participants with suicidal ideation were less likely to be partnered or have such information.

### Mental health outcomes in participants with and without suicidal ideation

Prevalence rates for somatic symptoms, symptoms of depression, and symptoms of PTSD according to cut-off scores as well as mean scores for self-rated mental and physical health, prevalence for experiences of sexual violence, combat or exposure to a war-zone and captivity are displayed in total and stratified by participants with and without suicidal ideation in Table 2.

**Table 2:** Prevalence of somatic symptoms, symptoms of depression, and symptoms of PTSD as well as self-rated mental and physical health status, experiences of sexual violence, combat or exposure to a war-zone and captivity in recently arrived refugees in Germany stratified by suicidal ideation

	Participants with suicidal ideation n/N (%)	Participants without suicidal ideation n/N (%)	***χ***^***2***^	Total n/N (%)
**Somatic symptoms**				
SSS-8 cut off > 11	84/168 (50%)	89/389 (22.9%)	40.303*	173/557 (31.1%)
**Symptoms of Depression**				
PHQ-8 cut off > 9	121/170 (70.8%)	100/393 (25.4%)	104.077*	221/563 (39.3%)
**Symptoms of PTSD**				
PCL-5 cut-off > 32	110/167 (65.9%)	88/388 (22.4%)	94.892*	198/555 (35.7%)
**Experiences of sexual violence**				
**yes**	87/170 (51.2%)	117/390 (30%)	22.926*	204/560 (36.4%)
**Combat or exposure to a war-zone**				
**yes**	59/171 (34.5%)	117/393 (29.8%)	1.243	176/564 (31.2%)
**Captivity**				
**yes**	75/171 (43.9%)	144/393 (36.6%)	2.614	219/564 (38.8%)
	**M / SD**	**M / SD**	***t***	**Total M / SD**
**Self-rated mental Health**				
range 0–100	28.22 / 30.72	53.86 / 33.29	8.851*	46.08 / 34.58
**Self-rated physical Health**				
range 0–100	40.29 / 32.67	62.29 / 31.49	7.536*	55.61 / 33.40

*p < .001

Compared to participants without suicidal ideation, participants with suicidal ideation reported significantly higher rates for somatic symptoms (*χ*^*2*^(1, 557) = 40.303, *p* < .001; 50% vs. 22.9%), depression (*χ*^*2*^(1, 563) = 104.077, *p* < .001; 70.8% vs. 25.4%), and PTSD (*χ*^*2*^(1, 555) = 94.892, *p* < .001; 65.9% vs. 22.4%). Moreover, participants with suicidal ideation more often reported experiences of sexual violence (*χ*^*2*^(1, 560) = 22.926, *p* < .001; 51.2% vs. 30%). In addition, significantly better self-rated mental (*t*(558) = 8.851, *p* < .001; 53.86 vs. 28.22) and physical health (*t*(561) = 7.536, *p* < .001; 62.29 vs. 40.29) were found in participants without suicidal ideation.

### Associations between suicidal ideation and socio-demographic and flight-related characteristics, different traumatic experiences as well as mental health outcomes

A logistic regression was run to test associations between suicidal ideation with different socio-demographic and flight-related characteristics (age, sex, university degree, partnership, current information about family left behind, and flight duration) experiences of captivity, war-zone exposure, and experiences of sexual violence as well as different mental health outcomes (symptoms of PTSD, depression, and somatoform symptoms) and self-rated mental and physical health status. The results are shown in Table 3.

**Table 3:** Logistic regression model for the association of suicidal ideation with different socio-demographic and flight-related characteristic as well as mental health outcomes in recently arrived refugees in Germany

Predictor	OR	95% CI	***p***
Age^1^	.**616**	**.440–.863**	***.005***
Sex^2^	1.345	.763–2.371	.305
University degree^3^	1.410	.862–2.304	.171
Partnership^3^	1.112	.673–1.838	.679
Current information about family members left behind^3^	.855	.522–1.402	.535
Flight duration^4^	**1.095**	**1.014–1.182**	**.020**
Experiences of sexual violence^5^	**2.253**	**1.355–3.748**	**.002**
Combat or exposure to a war-zone^5^	1.009	.588–1.732	.975
Captivity^5^	.707	.413–1.209	.205
Somatoform symptoms^6^	1.020	.977–1.064	.377
PTSD symptoms^7^	**1.143**	**1.009–1.041**	**.002**
Symptoms of depression^8^	**1.025**	**1.074–1.216**	**< .001**
Self-rated physical health^9^	.998	.989–1.008	.752
Self-rated mental health^9^	.990	.980–1.000	.056
Model fit indices:			
*χ*^*2*^ / df / *p* −2 Log-Likelihood	194.309 / 14 / < .001 435.823		
Nagelkerkes *R*^*2*^	.312		
Cox & Snell *R*^*2*^	.444		

^1^in 4 groups according to Table 1; ^2^female = 1, male = 2; ^3^yes = 1, no = 2; ^4^range 0–27; ^5^no = 0, yes = 1; ^6^Total score of SSS-8; ^7^Total score of PCL-5; ^8^Total score of PHQ-8; ^9^range 0–100

With respect to sociodemographic and flight related characteristics, younger age and longer flight duration were found to be associated with suicidal ideation and no associations were found between suicidal ideation and sex, university degree, partnership as well as current information about family members left behind. In addition, experiences of sexual violence and symptoms of PTSD and depression were found to be positively associated with suicidal ideation whereas no associations were found between suicidal ideation and combat experiences or exposure to war-zone, captivity and somatoform symptoms. All in all, the results suggest that significant associations between suicidal ideation and (I) younger age, (II) longer flight duration, (III) experiences of sexual violence, (IV) symptoms of PTSD, and (V) symptoms of depression are independent from one another.

## Discussion

Because only limited evidence is currently available concerning suicidal ideation among refugees hosted in Europe in recent years, in the present study, we examined the prevalence of suicidal ideation and its associations with different (1) socio-demographic characteristics, (2) flight and war-related traumatic experiences (e.g., combat-exposure, captivity), (3) experiences of sexual violence, and (4) different health outcomes in newly arrived refugees of different origins based on data collected at an initial reception facility in Leipzig, Germany.

In total, 171 participants (30.3%) reported having experienced suicidal ideation within the 2 weeks prior to the assessment, almost one third among them (*n* = 53; 30.9%) nearly every day. This finding converges with the results reported by Leiler et al. [18] who found that 33.9% of refugees residing in asylum accommodations in Sweden between November 2016 and April 2017, most of whom were from Afghanistan and Syria, experienced suicidal ideation. By contrast, Führer et al. [19] reported a 5.6% prevalence of suicidal thoughts as assessed via one item of HSCL-25 and based on a convenience sample (*n* = 209) of a heterogenous group of refugees living in Halle/Saale, Germany in August 2015. As Leiler et al. [18] used rather similar methods as the present study (e.g., sampling, assessment of suicidal ideation via item 9 of PHQ-9, participation of adult residents of asylum accommodation facilities), we assume that the results of the study presented here better reflect the actual prevalence of suicidal ideation among newly arrived refugees in Germany. However, since the evidence is quite limited to date, future research should investigate the prevalence of suicidal ideation among refugees residing in different European host countries more often and in more depth in order to provide an adequate database for drawing robust conclusions.

Regardless of the actual prevalence, however, the results of the present study clearly show the relationship between suicidal ideation and different mental health problems, including clinically relevant symptoms of PTSD and depression. In addition, participants reporting experiences of sexual violence seem to be at substantially increased risk of having suicidal ideation. Furthermore, the results of the regression analyses underline the independence of associations between suicidal ideation and experiences of sexual violence, symptoms of PTSD and depression, flight duration, and younger age, a finding that is in line with some previous research on suicidality among refugees [21–25]. In contrast to previous research findings indicating associations between suicidal ideation and war and/or displacement-related traumatic experiences [25, 26], no such associations were identified in the present study. That said, the proportion of participants who had experienced captivity and/or combat or exposure to a war-zone was quite high in the total sample, approximately one-third of all participants. All in all, the results emphasize the relationship between suicidal ideation and clinically relevant mental health symptoms among newly arrived refugees in Germany and require further and more in-depth research as there are only few comparable studies and, to the best of our knowledge, none at all on refugees currently living in Germany.

Although the present study has some major strengths – (1) epidemiological approach, (2) assessment of recently arrived refugees that accounts for the time frame of symptom burden and excludes long-term post-migration stressors, and (3) use of instruments that have been translated and back-translated into 11 different languages enabling participation of refugees from over 30 different countries – it also has a number of limitations that must be reflected on critically in order to shed light on specific implications for future research. First, the analyses of the present study are based on cross-sectional data and no information can be derived with respect to the causality of the associations analyzed because we are not able to determine the chronological sequence and long-term course of suicidal ideation, traumatic experiences, and mental health symptoms analyzed. Second, the findings limit the generalizability due to the fact that the data reflect a specific wave of refugees recently arrived in Germany at the time of data collection (e.g., refugees from Venezuela, Cameroon, and Syria) whereas the vast majority of refugees worldwide are located in camps within their countries of origin or bordering regions. Therefore, in the future, we need more research, especially using longitudinal approaches focused on suicidal ideation in different refugee populations hosted in different countries. Third, the findings are based on assessments of different mental health problems that were measured with self-rating instruments. Thus, future research is needed that uses different approaches of assessment to better understand the phenomenon and its clinical implications. Fourth, as did numerous studies before [18, 20, 32], the present study used only one item from the PHQ-9 to assess suicidal ideation. However, based on our current general understanding of suicidal ideation, no conclusions can be drawn about suicidal ideation without considering possible cultural and/or ethnic differences between the participants [33]. Thus, further research should focus in more detail on being culturally sensitive when assessing suicidal ideation. Beyond that, although some initial recommendations for addressing culture-specific features of suicidality among ethnic minorities and different groups of immigrants do exist [34], there is an urgent need for a specific theoretical framework explaining suicidality in refugees. Moreover, very little research is available on the protective factors reducing suicidality among refugees as well as specific, culture-sensitive prevention strategies for this highly burdened population [6].

## Conclusions

Despite the limitations and implications for future research mentioned above, the findings of the present study provide some first insights into how to establish and/or to improve health care services for newly arrived refugees experiencing suicidal ideation. First of all, mental and physical health professionals, different NGOs, and the staff members at initial reception facilities for newly arrived refugees should be properly trained based on available information on suicidal ideation among different refugee populations as well as prevention and intervention strategies in order to provide first interventions and to identify persons at risk. Additionally, special attention should be paid not only on refugees suffering from mental health symptoms, but also on younger people, refugees with longer flight duration and those who have experienced sexual violence, as these groups might be affected by suicidal ideation whether or not they have other mental health problems.

Ultimately, from our point of view, key parties at the political level who are responsible for managing the (health) care of refugees should be aware of the initial evidence-based insights of the phenomenon and reminded of their humanitarian obligations, so that the magnitude of the problem is recognized and understood at a broader community and societal level.

## Data Availability

The datasets generated and analyzed during the current study are not publicly available due to ongoing analyses in respect to other research questions, but are available from the corresponding author upon reasonable request.

## Abbreviations

**DSM**: Diagnostic and Statistical Manual of Mental Disorders
**LEC**: Life Event Checklist
**PCL**: PTSD Check List
**PHQ**: Patient Health Questionnaire
**PTSD**: Posttraumatic stress disorder
**UNHCR**: United Nations High Commissioner for Refugees
